# Advances in the Role of Adipose Tissue in Promoting Injury Repair and Resist Infection

**DOI:** 10.1002/iid3.70341

**Published:** 2026-02-04

**Authors:** Xi Duan, Run Li, Lei Fu, Jiali Yang, Zhean Zhan

**Affiliations:** ^1^ Pathology Department People's Hospital of Dayi County Chengdu China; ^2^ School of New Energy and Materials Southwest Petroleum University Chengdu China; ^3^ The People's Hospital of Renshou County Meishan China; ^4^ Mianyang Children's Hospital Mianyang China

**Keywords:** adipokines, adipose tissue, immune response, infection, injury repair, stromal, vascular components

## Abstract

**Background:**

In recent years, adipose tissue (AT) transplantation has increasingly been noticed by many people in the field of tissue repair and regeneration. Accumulating evidence demonstrates that AT exerts dual functions in promoting tissue repair and conferring anti‐infective properties, with distinct biological effects attributed to its heterogeneous components.

**Objective:**

This review systematically examines the distribution of AT and its components, including adipocytes, extracellular matrix (ECM), immune cells, stromal vascular fraction (SVF), and adipokines. Distinct AT components mediate tissue repair and infection resistance through unique molecular mechanisms.

**Results:**

Functionally, adipocytes and immune cells secrete various cytokines, including adiponectin, leptin, tumor necrosis factor‐α (TNF‐α), interleukin‐6 (IL‐6), antimicrobial peptides, and IL‐17, which coordinate inflammatory signaling and enhance host defense mechanisms. The main function of the ECM is to provide structural support for cells. SVF cell populations exhibit dual functionality: accelerating neural and cutaneous regeneration while suppressing fibrotic pathways to minimize scar formation.

**Conclusion:**

Recommendations are proposed to guide future investigations into AT‐mediated immune functions. This review highlights potential strategies for advancing AT‐based clinical therapeutics and suggests novel directions for foundational studies on AT's anti‐infective mechanisms.

## 1 Introduction

In mammals, AT comprises two principal subtypes: white adipose tissue (WAT) and brown adipose tissue (BAT), with WAT representing the predominant form. Anatomically, WAT is distributed in two primary depots: visceral depots surrounding intra‐abdominal organs and vasculature, and subcutaneous depots beneath the dermal layer [1, 2]. WAT primarily serves as the body's primary energy reservoir and a central regulator of metabolic homeostasis. Emerging evidence further reveals that WAT actively modulates immune responses through two key mechanisms: facilitating tissue repair processes and secreting immunomodulatory factors that assist immune cells in combating pathogens [3]. BAT demonstrates higher abundance in neonates and small mammals, where it facilitates thermoregulation under cold stress. In adults, BAT deposits are markedly reduced and predominantly localized to specific anatomical regions, including the interscapular, cervical, and paravertebral areas [4, 5]. Research on BAT has mostly focused on energy metabolism, and little is known about its effects on immune function. This review, therefore, concentrates on WAT due to its broader pathophysiological significance in adult populations.

Damage repair is a complex and dynamic process that necessitates the close coordination of multiple cells and factors to effectively restore damaged tissues [6]. Studies have demonstrated that AT plays a significant role in wound healing and infection resistance [3, 7, 8]. Evolutionarily conserved mechanisms observed in *Drosophila melanogaster* demonstrate adipocyte mobilization to wound sites, where they interact synergistically with platelets, erythrocytes, and macrophages to establish provisional wound closure, phagocytose cellular debris, and create antimicrobial barriers. Concurrently, adipocytes secrete (AMPs) that mediate direct bactericidal activity against invading pathogens. Concurrently, adipocytes secrete AMPs that mediate direct bactericidal activity against invading pathogens [3]. It has also been found that adipocytes have immune activity in mammals, and resident adipocytes in the skin can resist infection by *Staphylococcus aureus* [9]. Following dermal injury, myofibroblasts undergo adipogenic differentiation to support regenerative processes [10]. The cells in stromal vascular fraction (SVF) can activate macrophages, participate in immune regulation, and promote inflammation repair [11]. Collectively, AT functions as a multifunctional immunometabolic regulator, with distinct cellular constituents mediating compartmentalized functions across tissue repair cascades.

Existing research has largely focused on immunometabolic aspects, with most evidence concentrated on obesity‐related immunometabolic diseases [12, 13, 14, 15], systemic inflammatory states [16, 17], and the activation of pro‐inflammatory pathways [13]. While these studies have unveiled the human WAT atlas [18, 19] and immune cell subsets [16, 20, 21], and have mentioned intercellular interactions [19], they have not sufficiently clarified the roles of different tissue components and cell subpopulations in injury repair and immune defense. Moreover, the molecular drivers and regulatory mechanisms underlying the interactions among different cells during AT‐mediated injury repair remain inadequately explored. Therefore, this review systematically analyzes the roles of AT components in tissue repair and host defense. We elucidate the mechanisms by which different constituents function in infectious immunity, as well as the synergistic effects between adipokines and immune cells. By understanding the key mechanisms of AT in injury repair, we can develop adipose‐targeted strategies for regenerative medicine and the treatment of infectious diseases, thereby exploring new avenues for innovative therapies.

## 2 Anatomy and Physiology of AT

**Figure 1:**
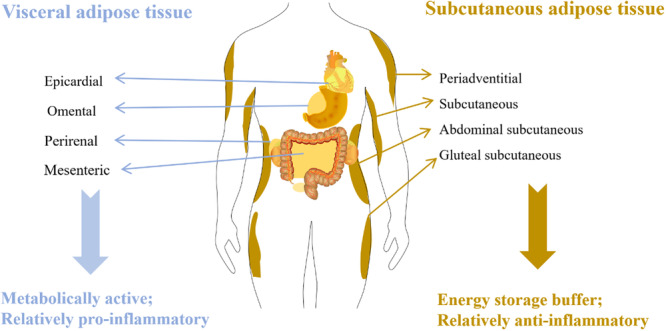
Diagram showing the distribution of visceral and subcutaneous fat in the human body.

In humans and other mammals, AT exhibits extensive anatomical distribution, with principal depots comprising subcutaneous compartments (superficial and deep abdominal layers in the upper body, gluteal‐femoral regions in the lower body) and visceral compartments including the omentum, mesentery, mediastinum, gonadal, and epicardial regions [22]. Subcutaneous adipose tissue (SAT), the most prevalent AT subtype, primarily fulfills thermoregulatory, mechanical cushioning, and energy storage roles [23] (Figure 1). In contrast, visceral adipose tissue (VAT) surrounding internal organs exhibits strong pathological associations with metabolic dysregulation, including diabetes mellitus and cardiovascular pathologies [24] (Figure 1), mediated through visceral organ compression and endocrine disruption. Sex‐specific body fat distribution patterns emerge in adulthood: females exhibit SAT predominance with gluteofemoral fat deposition (“pear‐shaped” morphology), whereas males demonstrate VAT predominance characterized by central adiposity (“apple‐shaped” morphology) surrounding abdominal viscera [25, 26, 27, 28].

AT is not merely a passive energy depot but a complex, dynamic immunometabolic organ [29]. Its physiological architecture is intricately designed to support the organism in responding swiftly to injury or pathogen invasion. Cellular and stromal composition as an immunogenic niche: AT comprises cellular and structural constituents—adipocytes, the extracellular matrix (ECM), vascular networks, diverse immune populations, neural innervation, and the SVF [30, 31, 32]—which collectively create a unique microenvironment permissive for immune communication and response.

White adipocytes, the predominant cellular component of AT, feature characteristic unilocular lipid droplets and sparse mitochondrial content [33]. beyond storing energy, function as sentinel cells that can rapidly respond to metabolic stress or infection by secreting signaling molecules. Additionally, upon injury, adipocytes can actively migrate to the wound site and collaborate with macrophages to establish a barrier against bacteria [3]. ECM of AT constitutes a dynamic scaffold comprising collagenous proteins, glycoproteins, glycosaminoglycans, and proteoglycans [34]. This biomechanical framework ensures adipocyte spatial organization while maintaining tissue structural integrity through tensile strength and elastic recoil properties [35]. Beyond structural roles, the ECM orchestrates bidirectional signaling via integrin‐mediated adhesion complexes and growth factor sequestration/release (e.g, transforming growth factor [TGF]‐β, VEGF), dynamically coordinating adipogenic programming and metabolic homeostasis [36]. The dense vascular network is crucial for delivering nutrients [37] and serves as the primary conduit for immune cell recruitment from the circulation to AT in response to local signals. Crucially, AT constitutively harbors resident immune populations, notably macrophages and lymphocytes. These immune cells coordinate immune metabolic homeostasis by dynamically regulating inflammatory tension and tissue remodeling processes [38]. SVF: SVF comprises endothelial cells, vascular smooth muscle cells, progenitor cells, and multipotent mesenchymal stromal cells [39]. The mesenchymal stromal cells and preadipocytes possess immunomodulatory properties that influence the behavior of neighboring immune cells [40]. The regenerative potential of SVF predominantly resides in preadipocytes—adipocyte precursors with mesenchymal lineage plasticity and multilineage differentiation capacity [41, 42]. Therefore, it can be applied to tissue repair and functional reconstruction. Adipokines [43]: the endocrine‐immune bridge: this physiological infrastructure enables AT to function as a potent endocrine and paracrine organ. It secretes an array of adipokines—such as leptin, adiponectin, and interleukin (IL)‐10—that directly mediate immune crosstalk [44]. For instance, leptin modulates immune cell activity [45], while adiponectin exerts anti‐inflammatory effects [46, 47]. Furthermore, AT can produce AMPs, bridging its metabolic identity with direct host defense [3].

Therefore, the very physiological design of AT—its cellular diversity, specialized stroma, and secretory profile—establishes the foundation for its emerging roles in tissue repair and anti‐pathogen defense. The following sections will dissect how these individual components (the adipocytes, ECM, adipokines, and specific immune cells within the SVF) act in concert to promote wound healing and resist pathogen infection.

## 3 The Role of Different Components

### 3.1 The Role of Different Components: Adipocyte

White adipocytes exhibit a characteristic unilocular morphology, with a singular lipid droplet occupying approximately 90% of the cytoplasmic volume [48]. This lipid droplet is enveloped by a phospholipid‐cholesterol monolayer membrane that stabilizes neutral lipid stores. The displaced nucleus, compressed peripherally by the lipid droplet, constitutes merely 2%–3% of total cellular volume [49, 50]. The adipocyte plasma membrane harbors specialized receptors (e.g., insulin receptor, leptin receptor) that mediate metabolic regulation and endocrine signaling [51].

**Figure 2:**
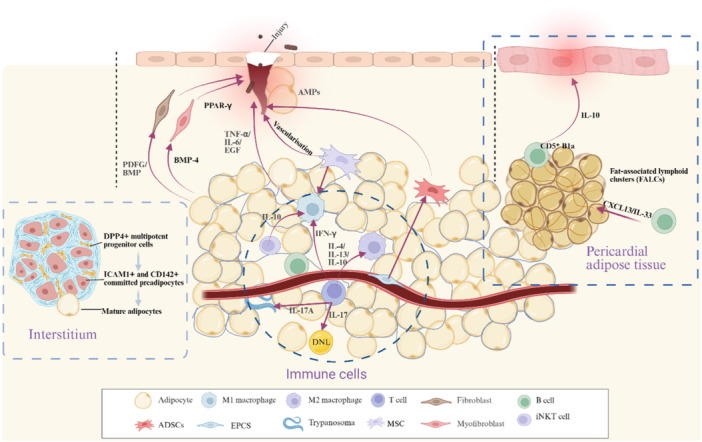
The role of different components in AT in repair and anti‐infection. In *Drosophila*, adipocytes exhibit actin‐dependent non‐adherent migration to wound sites during tissue repair and simultaneously secrete AMPs. Adipocyte‐mediated fibroblast activation may result from the actions of PDGF and BMP signaling molecules. Adipocyte‐derived BMP‐4 induces myofibroblast activation and stimulates PPAR‐γ activity. The EMC primarily provides support for cells in AT, acting as a reticulated interstitium that contains DPP4⁺ preadipocytes, which subsequently differentiate into ICAM1⁺ and CD142⁺ precursor adipocytes before maturing into adipocytes. M1 macrophages in AT secrete pro‐inflammatory factors such as TNF‐α and IL‐6 to participate in inflammatory responses, while M2 macrophages secrete anti‐inflammatory factors, including IL‐10 and TGF‐β. γδ T cells secrete IL‐17, which can mediate immune responses and also control local parasite load. T cells promote M1 polarization by secreting cytokines such as IFN‐γ, whereas IL‐4, IL‐13, and IL‐10 drive M2 differentiation. iNKT cells can produce IL‐10 to suppress macrophage infiltration in AT. In pericardial AT, B1a cells reside within FALCs; CD5⁺ B1a cells migrate to the heart and promote inflammation resolution and injury repair via IL‐10 secretion. EPCs in the SVF can promote angiogenesis through paracrine signaling, and VC05 can activate preadipocytes. MSCs mediate the recruitment of macrophages, keratinocytes, and fibroblasts, and release EGF and TGF‐α to promote wound healing; they also induce angiogenesis. ADSCs migrate to wound sites, differentiate into endothelial cells and fibroblasts, and release angiogenic factors through paracrine mechanisms.

Emerging evidence demonstrates that AT plays an active regulatory role in cutaneous repair processes. Franz et al. demonstrated through *D. melanogaster* models that adipocytes exhibit actin‐dependent non‐adhesive migration toward wound sites during tissue repair [3]. These mobilized adipocytes coordinate with macrophages to facilitate cellular debris clearance and restore epithelial continuity, concurrently secreting AMPs to establish antimicrobial barriers against invading pathogens (such as *Escherichia coli*) [3]. During the acute phase of cutaneous wound healing, adipocyte lineage cells become functionally activated [8, 52, 53]. While adipocyte‐mediated fibroblast activation is essential during the proliferative phase of tissue repair, the precise molecular crosstalk between these cell types remains incompletely characterized. Current hypotheses suggest adipocyte‐derived signaling molecules, particularly platelet‐derived growth factor (PDGF) and bone morphogenetic protein (BMP), may mediate these regulatory interactions [8]. Lineage tracing studies employing *Sm22‐Cre* and Sma‐CreER murine models reveal myofibroblast‐derived adipogenesis contributes significantly to neoadipocyte populations in regenerating wounds [10]. Furthermore, Hoerst et al. demonstrated that adipocyte‐derived BMP‐4 induces myofibroblast activation and subsequent secretion of paracrine mediators that stimulate PPAR‐γ‐dependent anti‐fibrotic activity, effectively attenuating pathological scar formation [54] (Figure 2). Mice with adipocyte‐specific nuclear corepressor (NCoR) ablation demonstrated significant protection from experimental skin fibrosis and inflammation. The protective effects were mediated primarily through endogenous PPAR‐γ [55].

### 3.2 The Role of Different Components: ECM

ECM refers to the three‐dimensional (3D) network structure outside cells, primarily composed of proteins, glycoproteins, glucuronic acid, and polysaccharides [56]. ECM provides mechanical support and structural framework for adipocytes, essential for maintaining the shape and integrity of AT [57]. ECM also plays important functional roles in interacting with numerous growth factors and signaling molecules to regulate cellular events such as cell adhesion, proliferation, migration, survival, and differentiation [36]. Kim et al. found that when using ECM for tissue engineering scaffolds, cytokine production was observed in vitro, and when applied to rat wounds, it accelerated wound healing, improved epithelialization, increased dermal thickness, collagen deposition, and vascular distribution [58]. Changes in the components of the ECM can affect the growth, differentiation, and apoptosis of adipocytes, thereby regulating the development and metabolic state of AT. ECM also plays a crucial role in angiogenesis, promoting the formation of new blood vessels to provide necessary oxygen and nutrients for adipocytes [59], supporting the metabolic needs of AT. Lee et al. demonstrated that human AT‐derived ECM wound dressings promote superior healing outcomes versus conventional treatments, exhibiting structurally organized granulation tissue and increased microvascular density indicative of robust angiogenesis [60]. Human AT‐derived ECM potently enhances wound repair by driving re‐epithelialization and neovascularization, while optimizing collagen fibril alignment and maturation‐phase matrix deposition. The effectiveness of wound healing is significantly influenced by the interaction between ECM components and cells, as well as growth factors, a dynamic and reciprocal process. In chronic wounds, the normal healing process is delayed due to underlying systemic dysfunction. When these wounds exhibit ECM defects or functional impairments, characterized by ECM distortion, they will fail to support wound healing [61, 62, 63].

The ECM also serves as a reservoir for adipocytes. DPP4^+^ [64] progenitors reside in the reticular interstitium and give rise to committed preadipocytes. These DPP4+ multipotent progenitor cells differentiate into ICAM1+ and CD142+ committed preadipocytes, which are poised to mature into adipocytes. While committed preadipocytes are intercalated between mature adipocytes and occupy a perivascular niche, DPP4+ progenitors are located within the reticular interstitium [65, 66], a recently recognized but still not well‐studied, fluid‐filled network of collagen and elastin fibers that surrounds many organs, including adipose depots. It can thus be inferred that the reticular interstitium corresponds to the ECM and contains progenitor cells capable of differentiating into mature adipocytes, providing lineage‐tracing evidence for the cellular origin of adipocytes (Figure 2).

### 3.3 The Role of Different Components: Immune Cells

AT functions not only as an energy reservoir but also harbors diverse immune cell populations that orchestrate immunometabolic homeostasis. Macrophages play a crucial role in AT, participating in inflammatory responses and fat metabolism, and can differentiate into M1 (pro‐inflammatory) and M2 (anti‐inflammatory) [67]. M1 macrophages produce pro‐inflammatory cytokines (such as tumor necrosis factor‐α [TNF‐α], IL‐6), which are involved in inflammatory responses. Classically activated M1 macrophages, often identified by CD11c [68] expression, accumulate around necrotic adipocytes and secrete excessive pro‐inflammatory cytokines like IL‐6 and TNF‐α, a hallmark in both human obese AT and murine models [69, 70]. Whereas M2 macrophages contribute to tissue repair and anti‐inflammatory reactions [71]. Alternatively activated M2 macrophages (F4/80+CD11c−CD206+) found in lean AT produce substantial anti‐inflammatory cytokines such as IL‐10 [68, 72] (Figure 2). In lean physiological states, the main components are eosinophils, M2‐like anti‐inflammatory macrophages, and innate lymphocytes, which maintain metabolic homeostasis within AT. In the process of obesity, pro‐inflammatory immune cells (such as M1‐like macrophages) dominate in AT [73, 74]. The immune characteristics of AT are characterized by the recruitment of macrophages, neutrophils, and cytotoxic CD8^+^ T cells, leading to a pro‐inflammatory state [75, 76]. While the importance of macrophages and their interaction with adipocytes and other immune cells is now accepted as key in the development of adipocyte and metabolic dysfunction, the mechanisms regulating these interactions are not well understood.

Recent studies have further revealed the critical regulatory roles of specific T‐cell subsets and cytokine signaling within the AT immune microenvironment in metabolic homeostasis and infection response. Among these, γδ T cells and the IL‐17 signaling pathway play important roles in maintaining AT function and mediating de novo adipogenesis [77, 78, 79, 80]. IL‐17 exhibits circadian fluctuations, and its signaling directly regulates de novo lipogenesis (DNL) in adipocytes via the IL‐17 receptor, thereby influencing lipid storage and metabolic balance [80]. In infection models, IL‐17 signaling contributes significantly to AT responses [81, 82, 83]. For instance, during *Trypanosoma brucei* infection [84], adipocyte‐intrinsic IL‐17A signaling participates in controlling local parasite burden [83], while IL‐17A/F drives epididymal WAT wasting and lipid utilization in male mice, indicating a sex‐dimorphic metabolic regulatory function [85]. Furthermore, distinct T‐cell subsets exert stage‐specific effects on infection‐induced AT wasting. In the early phase of *T. brucei* infection, CD4⁺ T cells are the key population inducing AT atrophy and are closely associated with anorexia‐related fat loss, whereas during later stages, CD8⁺ T cells become the primary drivers of muscle atrophy and sustained AT depletion [86]. In terms of immune cell interactions, T cells can regulate macrophage phenotypes by secreting cytokines, such as IFN‐γ, promoting pro‐inflammatory M1 polarization, while IL‐4, IL‐13, and IL‐10 drive anti‐inflammatory M2 differentiation, thereby affecting the inflammatory state of AT [70, 87]. Additionally, studies have shown that invariant natural killer T (iNKT) cells [88] can suppress macrophage infiltration in AT, improve adipocyte insulin sensitivity, and elevate adiponectin levels through the production of anti‐inflammatory factors such as IL‐10, highlighting their unique role in immunometabolism [89]. In summary, γδ T cells, IL‐17 signaling, and various T‐cell subsets (CD4⁺, CD8⁺) within AT not only participate in metabolic regulation under basal conditions but are also deeply involved in immunometabolic remodeling and wasting pathologies during infections and other stress conditions. These insights provide a new and important dimension for understanding the crosstalk between immunity and metabolism (Figure 2).

Although B cells are present at low levels in AT [18], they play important roles in tissue repair and immune regulation. Studies have found that pericardial AT is enriched with such B‐1a cells [90], which reside in fat‐associated lymphoid clusters (FALCs). Their recruitment and maintenance rely on the chemokine CXCL13 and the cytokine IL‐33 expressed by adipose stromal cells [91, 92]. In an acute myocardial infarction model, CD5⁺ B‐1a cells in pericardial fat expand and migrate to the heart, where they promote inflammation resolution and improve cardiac function and injury repair by secreting IL‐10; loss of B‐cell‐derived IL‐10 exacerbates myocardial injury and delays repair [93]. B‐1 cells belong to the innate‐like B‐cell lineage, exhibit strong TLR responsiveness, and are not only present in the peritoneal cavity but also represent a major B‐cell subset in VAT [91, 94]. FALCs, as tertiary lymphoid structures associated with visceral fat, are rich in B cells [95] and can support B‐cell proliferation, differentiation into plasma cells, and antibody production upon inflammatory stimulation [96]. Their formation depends on myeloid‐derived TNF and stromal TNFR signaling, and requires the involvement of CD1d‐restricted NKT cells and IL‐13 [97]. At the metabolic and immunoregulatory level, B cells in AT exert their functions through multiple mechanisms, including antigen presentation, cytokine secretion, and antibody production. In early obesity, IgG⁺CD19⁺ B cells increase in AT; B‐cell deficiency reduces M1‐type macrophage infiltration and CD8⁺ T‐cell‐mediated IFN‐γ expression, thereby improving glucose tolerance [98]. IgG produced by B cells can promote the clearance of apoptotic debris through complement activation, while regulatory B cells (Breg) secrete IL‐10, which plays an anti‐inflammatory role in suppressing AT inflammation [98] (Figure 2).

Immune cells in AT play a crucial role in maintaining immune balance, regulating metabolism, and participating in inflammatory responses. Current research predominantly targets the metabolic regulatory functions of AT innate immune cells, while their contributions to tissue repair remain underexplored. The response mechanisms of AT innate immune cells to pathogenic challenges constitute a significant knowledge gap demanding further mechanistic exploration.

### 3.4 The Role of Different Components: SVF

SVF denotes the cell population extracted from AT, comprising preadipocytes, immune cells, endothelial cells, smooth muscle cells, fibroblasts, and macrophages [30, 99, 100]. When integrating vascular cells in AT, Massier et al. found that endothelial precursor cells (EPCs) are enriched for genes such as TYROBP and FCER1G, representing a hematopoietic cell type that promotes angiogenesis through paracrine mechanisms [101]. Another vascular subtype (vC05) expresses multiple marker genes that activate preadipocytes (e.g., CXCL14, APOD, and CFD) and is the only vascular population expressing PDGFRA [101]. In mice, all periaortic mural fibroblasts are PDGFRA⁺ and give rise to perivascular adipocytes [102]. Among EPCs, one subtype—early EPCs—shows high expression of transcripts specific to hematopoiesis (RUNX1, WAS, LYN) and is associated with immune and inflammatory functions (e.g., TLRs, CD14, HLAs) [103]. Another subtype, outgrowth endothelial cells, displays prominent expression of transcripts involved in vascular development and angiogenic signaling pathways (Tie2, eNOS, Ephrins) [103]. Thus, EPCs within AT vascular cells can promote vessel formation and development in response to injury, while the vascular subtype termed vC05 may represent an intermediate cell state between endothelial cells and adipocyte precursors [101] (Figure 2). SVF not only maintains AT function and overall metabolism but also plays a crucial role in promoting tissue repair and anti‐fibrosis. Furthermore, the combination of SVF cells and adipose‐derived stem cells (ADSCs) exhibits immunomodulatory and anti‐inflammatory effects, which secrete anti‐inflammatory factors that ameliorate function in autoimmune encephalomyelitis models from both immune and pathological perspectives [11]. Further research demonstrates that SVF activates macrophages, reduces inflammatory cell infiltration, and mitigates neuroinflammation in tissues; it also upregulates the level of the anti‐inflammatory cytokine IL‐10 in the spleen; SVF cells rapidly and effectively dampen the spread of autoimmune signaling in peripheral tissues. Choi et al. treated liver injury with SVF and demonstrated that it inhibits TAA‐induced liver fibrosis by secreting IL‐10 and repairs liver function by suppressing inflammatory cell infiltration and promoting capillary and hepatocyte regeneration in vivo [104]. Nan's demonstrated that SVF gel promotes wound healing and peripheral nerve repair in diabetic rats, possibly through the activation of TLRs/MyD88/NF‐κB signaling pathways [105]. Clinical studies indicate that SVF injection accelerates primary skin wound healing and improves scar appearance over a 6‐month follow‐up [106].

The regenerative capacity of AT is primarily attributed to the precursor cells in SVF, ADSCs, which are pluripotent stem cells isolated from it [107]. Due to their advantages, such as stable proliferation in vitro, low apoptosis rate, easy accessibility, and suitability for autologous transplantation [108], they have gradually become one of the new research hotspots in recent years, widely applied in tissue engineering, especially in injury repair. ADSCs are mesenchymal stem cells (MSCs) characterized by their presence in the basal layer of hair follicles, the dermis [109], and visceral WAT [110, 111], as well as by their ability to proliferate and differentiate into skin cells for cellular repair [105, 112]. MSCs play a crucial role in wound healing and tissue repair [113, 114]. They manipulate macrophages to recruit keratinocytes and fibroblasts; these macrophages release epidermal growth factor (EGF) and TGF‐α to stimulate keratinocyte migration and proliferation, thereby aiding wound repair [115]. Additionally, MSCs can induce angiogenesis [116, 117] and promote the recovery of neural function [118, 119]. Extracellular vesicles derived from MSCs can effectively overcome issues such as host rejection in skin repair therapies and exhibit multiple therapeutic effects on skin regeneration [120, 121]. Beyond their role in skin injury repair, MSCs also promote wound healing in diabetic feet [122], repair of liver injury [123], and cardiac tissue repair [124]. IL‐17A and IL‐17F treatment of 3T3‐L1 pre‐adipocytes inhibited adipogenesis, leading to reduced production of leptin [125]; this may, in turn, affect inflammation within AT (Figure 2). ADSCs also activate the cell regeneration and healing process through autocrine and paracrine pathways. During wound healing, ADSCs exhibit strong migratory capabilities, quickly being recruited to the injured site, and can differentiate into dermal fibroblasts (DFs), endothelial cells, and keratinocytes [109]. ADSCs and DFs are the primary sources of ECM proteins involved in maintaining skin structure and function [126]. Encapsulating ADSC exosomes hADSCs‐Exos in hydrogel PF‐127 can effectively promote the healing of large‐area skin injuries [127]. In murine burn models, ADSC‐enriched hydrogels significantly accelerate wound closure and facilitate scarless tissue regeneration through multifaceted mechanisms; specifically, they evade immune recognition and activation, thereby reducing inflammation, promoting collagen deposition and angiogenesis, and enhancing M2 macrophage polarization in wounds [128]. ADSCs also inhibit granulosa cell apoptosis and senescence, thereby preventing chemotherapy‐induced premature ovarian failure. They improve the ovarian microenvironment damaged by cyclophosphamide (CTX), increase follicular cell numbers, and enhance estradiol secretion [129]. Furthermore, ADSCs promote angiogenesis via direct differentiation into endothelial cells and paracrine release of angiogenic factors [130, 131, 132].

The immunomodulatory, angiogenic, and anti‐fibrotic properties of the nutritional factors derived from SVF and ASC can be used to promote repair and improve scar formation while reducing inflammatory responses. dECM hydrogels containing SVF have demonstrated feasibility for preclinical use [133]. Additionally, other functions of SVF ensure the supply of nutrients (such as glucose and fatty acids) and oxygen, meeting the metabolic needs of adipocytes. Multiple hormones secreted by AT are transported throughout the body via the circulatory system through vascular components. The vascular structure of AT allows immune cells (e.g., macrophages and T cells) to migrate into AT, participating in local immune responses and inflammatory processes.

### 3.5 The Role of Different Components: Adipokines

In addition to storing energy in the form of lipids, the AT is considered an important endocrine organ capable of secreting various factors that play a crucial role in regulating energy metabolism, inflammatory responses, and other physiological processes. Key secreted mediators encompass: (1) Metabolic hormones (leptin, adiponectin) and growth factors [134, 135]; (2) Immunomodulators, including pro‐inflammatory (IL‐6, IL‐1β, TNF‐α) and anti‐inflammatory mediators; (3) Complement components [136]; (4) AMPs exhibiting direct bactericidal activity [3, 9]. These factors released by the AT are collectively known as adipokines.

**Figure 3:**
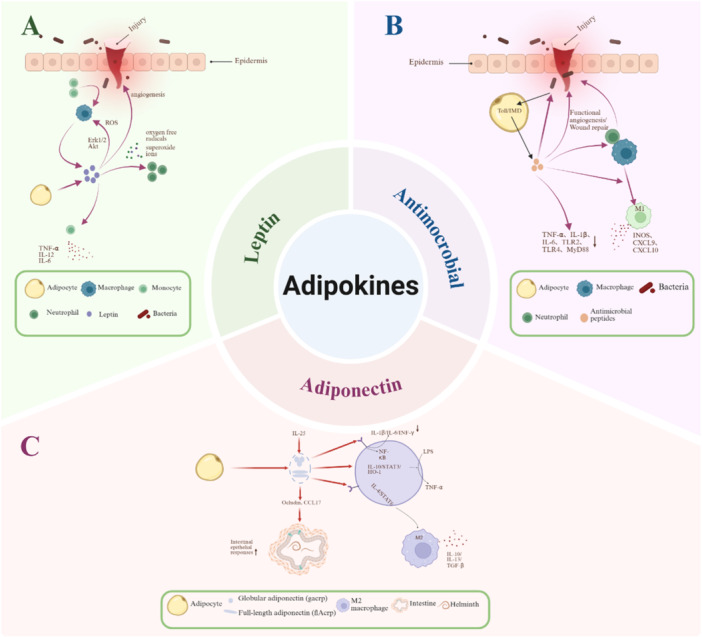
The role of adipokines in injury repair and immunity. (A) Regulates neutrophil activity and function by increasing chemotaxis and the secretion of oxygen‐free radicals and superoxide ions. Leptin stimulates monocytes to produce TNF‐α, IL‐12, and IL‐6, enhancing phagocytosis and the secretion of inflammatory mediators. Leptin can promote angiogenesis, activate the PI3K and MAPK pathways to inhibit apoptosis, and also stimulate the production of ROS in macrophages via Erk1/2 and Akt phosphorylation, aiding in phagosome formation and the oxidative killing of parasites. (B) β‐defensin and LL‐37 can directly kill bacteria, viruses, and fungi, and can also recruit macrophages and neutrophils to the site of infection. They can downregulate the expression of pro‐inflammatory cytokines (TNF α, IL 1β, IL 6), Toll‐like receptors (TLR2 and TLR4), and their downstream molecule MyD88 in chicken serum and the empty field. LL‐37 can directly promote the differentiation of macrophages into the pro‐inflammatory M1 phenotype, characterized by the production of iNOS, CXCL9, and CXCL10. Microbial infections activate the Toll and IMD signaling pathways in the *Drosophila* fat body, thereby triggering the expression and secretion of AMPs. (C) AdipoR1 mainly binds gacrp to inhibit macrophage NF‐κB activation and pro‐inflammatory factor expression, while gacrp activates the IL‐10/STAT3/HO‐1 pathway to suppress TNF‐α expression in LPS‐stimulated Kupffer cells. flAcrp promotes M2 polarization via AdipoR2 through the IL‐4/STAT6 pathway. IL‐25 regulates adiponectin to upregulate the expression of Occludin and CCL17 to facilitate worm expulsion.

Leptin is a hormone secreted by adipocytes, serving as a bridge between nutritional status and the immune system in the body [137]. In innate immunity, leptin regulates neutrophil activity and function by increasing chemotaxis and the secretion of oxygen‐free radicals and superoxide ions [138]. Additionally, leptin stimulates monocytes to produce TNF‐α, IL‐12, and IL‐6, thereby enhancing monocytes' phagocytosis, promoting pro‐inflammatory mediator secretion during acute‐phase responses, and upregulating adhesion molecule expression [139, 140] (Figure 3A). When the skin is infected with *S. aureus*, monocytes transform into macrophages. After several weeks of persistent infection, macrophages regulate the expansion of subcutaneous adipocytes and the production of adipokine hormones such as leptin, which controls angiogenesis and reconstructs wound repair after infection [141]. Leptin responds positively to pathogen invasion by modulating multiple immune cell types. During inflammation, leptin stimulates macrophage and neutrophil recruitment in vivo; in vitro, it activates PI3K and MAPK signaling pathways to suppress apoptosis [142] (Figure 3A). Leptin stimulates macrophages by inducing the phosphorylation of Erk1/2 and Akt, while also enhancing intracellular ROS generation to induce macrophage phagocytic activity, which aids in the formation of phagosomes and oxidative killing of parasites [143] (Figure 3A).

Adiponectin is a key regulator of innate immunity that modulates inflammation and metabolic disorder progression. It exerts pleiotropic effects by binding to adiponectin receptors 1 and 2 (AdipoR1/AdipoR2) [144]. AdipoR1 primarily binds globular adiponectin (gacrp), mediating inhibition of NF‐κB activation and pro‐inflammatory cytokine expression in macrophages [145]. Gacrp inhibits TNF‐α expression in Kupffer cells stimulated by LPS through activating the IL‐10/STAT3/HO‐1 pathway [146]. Full‐length adiponectin (flAcrp) shifts macrophages toward an M2 phenotype via AdipoR2 in an IL‐4/STAT6‐dependent manner [147] (Figure 3C). Adiponectin initially increases TNF‐α production by macrophages via ERK1/2→Egr‐1 and NFκB‐dependent mechanisms; these increases in TNF‐α in turn lead to increased expression of IL‐10 and an eventual dampening of LPS‐mediated cytokine production in macrophages [148, 149, 150]. Beyond inflammatory regulation, adiponectin also contributes to parasite clearance. Studies demonstrate that IL‐25‐induced adiponectin upregulates Occludin and CCL17 expression, enhancing intestinal epithelial responses to promote helminth expulsion and barrier integrity [151] (Figure 3C). Concurrently, key cytokines exhibit multifaceted roles: TNF‐α (pro‐inflammatory) promotes adipocyte apoptosis while driving inflammation; IL‐6 exerts context‐dependent pro‐ or anti‐inflammatory effects; Monocyte chemoattractant protein‐1 (MCP‐1) recruits monocytes to AT, amplifying inflammatory cascades.

In addition to the aforementioned factors, recent studies have found that AT can secrete AMPs, playing a crucial role in regulating immune responses and defending against microbial infections. The AT can synthesize and secrete various AMPs, such as β‐defensins (β‐defensin) and LL‐antimicrobial peptides (LL‐37) (Figure 3B). These AMPs exhibit broad‐spectrum antibacterial activity, capable of directly killing bacteria, viruses, and fungi, contributing to the host's defense mechanisms. Some of these AMPs, such as defensins and cathelicidins, have chemotactic properties and recruit immune cells, such as macrophages and neutrophils, to the site of infection [152, 153]. They are also capable of downregulating the gene expression of pro‐inflammatory cytokines (TNF‐α, IL‐1β, IL‐6), Toll‐like receptors (TLR2 and TLR4), and their downstream molecule MyD88 in the serum and jejunum of broilers [154, 155]. Cathelicidin‐related AMP or LL‐37 has also been reported to modulate cytokine and chemokine production, apoptosis, functional angiogenesis, or wound repair by stimulating keratinocyte migration and proliferation [156]. Moreover, LL‐37 can directly promote the differentiation of macrophages into the pro‐inflammatory M1 phenotype, characterized by the production of iNOS, CXCL9, and CXCL10 [157] (Figure 3B). Buchon demonstrated that microbial infections activate Toll and IMD signaling pathways in the fat body, triggering systemic expression and secretion of AMPs [158]. Franz also found that the fat body of fruit flies can release AMPs at wound sites to combat infection with [3], noting that the dynamic changes in this localized antimicrobial effect of AT in vertebrate wounds require further research. Consequently, recent studies have found that mouse adipocytes can produce AMP after bacterial skin infections [9].

Adipokines exhibit multifaceted functions—including anti‐inflammatory, immunomodulatory, and antioxidant properties—that significantly contribute to host defense against infections. In‐depth investigation of adipokine‐mediated immune responses holds promise for developing novel anti‐infective therapies and mitigating infection risks in patients with metabolic disorders.

## 4 Comparative Analysis of Immune Microenvironment and Cellular Interactions in SAT and VAT

There is extensive intercellular communication between immune and non‐immune cells in AT. This crosstalk is mediated through secretory factors (such as cytokines and adipokines) and direct contact, collectively regulating both local and systemic metabolic and immune homeostasis [159]. This crosstalk is mediated through secretory factors (such as cytokines and adipokines) and direct contact, collectively regulating both local and systemic metabolic and immune homeostasis [159, 160]. SAT and VAT differ in cellular composition, immune cell infiltration patterns, and microenvironmental signaling, leading to distinct modes and outcomes of intercellular communication between the two depots [161, 162, 163, 164].

The communication between immune cells and non‐immune AT cells exhibits distinct features in SAT and VAT. VAT demonstrates a more pronounced pro‐inflammatory immune cell infiltration and activation state [162, 165]. Its immune infiltration is primarily dominated by myeloid cells (e.g., M0 macrophages, monocytes, and neutrophils) and shows positive correlations with monocytes, M1‐type macrophages, and activated mast cells, while negatively correlating with M2‐type macrophages and resting mast cell states [166]. VAT also contains a higher proportion of pro‐inflammatory T helper cells (Th1, Th17) and CD8⁺ T cells compared to SAT [167]. These immune cells secrete cytokines such as TNF‐α, IL‐6, and IL‐13 [168], which interact with adipocytes to exacerbate local inflammation and insulin resistance [169, 170]. Concurrently, upregulated genes in VAT are enriched in signaling pathways including MAPK, PI3K‐Akt, and IL‐17, which are involved in inflammatory responses, cell survival, and immune regulation, thereby further reinforcing pro‐inflammatory intercellular communication [166].

In contrast, SAT generally exhibits relatively anti‐inflammatory and metabolically healthy communication characteristics. SAT shows a stronger correlation with leptin [171], and its shared hub genes are negatively associated with M1 macrophages and lymphocytes. SAT has a more pronounced influence on memory B‐cell activity [172], with B cells participating in immune regulation through antibody production, antigen presentation, and cytokine secretion [173]. In terms of signaling pathways, differentially expressed genes specific to SAT are primarily involved in cytokine‐cytokine receptor interaction, Toll‐like receptor signaling, and cell adhesion molecules [166], suggesting that its intercellular communication may be more focused on immune modulation and the maintenance of tissue architecture. Furthermore, intercellular communication in SAT is significantly influenced by estrogen, and estrogen deficiency can lead to increased VAT accumulation [174].

In summary, the intercellular communication between immune cells and non‐immune cells in SAT and VAT differs in cell types, polarization states, secretory factor profiles, and dominant signaling pathways. These distinct patterns of cellular crosstalk lead to divergent systemic metabolic effects of SAT and VAT. Due to its rich vascularization, stronger innervation, and greater sensitivity to catecholamine‐induced lipolysis, the pro‐inflammatory intercellular communication in VAT more readily contributes to systemic insulin resistance and metabolic syndrome [175]. Conversely, SAT, through its relatively anti‐inflammatory communication environment and its capacity to store free fatty acids [176], provides a degree of protection against metabolic disorders.

## 5 Clinical Significance

The clinical significance of AT lies in its dual functional nature: it serves as a crucial reservoir for tissue regeneration and repair, while simultaneously acting as a key driver of various chronic diseases. This duality stems from the inherent cellular heterogeneity of AT, its potent paracrine capacity, and its high plasticity in responding to local and systemic microenvironmental signals. Therefore, systematically elucidating the mechanisms and translational applications of AT in both “immune dysregulation leading to disease” and “promoting repair and regeneration” is essential for a comprehensive understanding of its clinical value and for developing targeted intervention strategies.

### 5.1 Role in Promoting Repair and Regenerative Medicine

AT plays a crucial role in regenerative medicine fields such as plastic and reconstructive surgery, stem cell therapy, and tissue engineering. Autologous fat transfer has various applications in plastic surgery, including breast reconstruction and remodeling [177], facial fat grafting [178], diabetic foot repair [179], and the treatment of lipoatrophy, muscular dystrophy, and other conditions [180, 181]. Autologous AT grafting represents a promising and safe approach for treating painful scars [182]. In reconstructive surgery involving autologous flap transplantation, macrophages within AT have been found to remodel collateral vessels, suggesting potential therapies targeting macrophages to enhance flap vascularization [183].

Recent advances in 3D culture technology have been pivotal for organoid cultivation. Organoids are complex 3D structures grown through the self‐organization of stem cells and exhibit functional and structural similarities to in vivo organs [184]. 3D bioprinting utilizing ADSCs has achieved initial success in constructing 3D tissues and organ reconstruction. For example, a 3D corneal‐like tissue was fabricated using ADSCs combined with embryonic stem cells via laser‐assisted bioprinting; after 7 days in porcine organ culture, ADSCs migrated from the printed structure [185]. 3D bioprinting of gelatin methacryloyl (GelMA) hydrogels incorporating ADSCs and human umbilical vein endothelial cells (HUVECs) can effectively improve the distribution of oxygen and nutrients within engineered tissues [186]. Encapsulating chondrocytes and adipocytes differentiated from ADSCs within hydrogels has been shown to enable ear regeneration [187].

ADSCs combined with suitable biomaterials exhibit potential for neural tissue regeneration. A composite scaffold of multi‐walled carbon nanotubes (MWNT) and polydimethylsiloxane (PDMS) implanted with differentiated ADSCs (dASCs), along with glial growth factors, promoted differentiation into Schwann cell‐like cells, indicating its applicability for peripheral nerve regeneration [188]. Adipose‐derived mesenchymal stem cells (ASCs) also participate in forming nascent vascular‐like structures [189], which are critical for treating post‐ischemic injury. ASCs isolated from the SVF of human AT expressing CD34, CD133, and ABCG2 have been shown to differentiate into endothelial cells and contribute to revascularization in murine ischemic hind limbs [130]. Furthermore, non‐adipocyte stromal cells within AT secrete a variety of angiogenic and anti‐apoptotic growth factors essential for promoting vascularization, such as VEGF and HGF, as well as PlGF, bFGF, angiopoietin, GM‐CSF, MCP‐1, and SDF‐1α [190, 191].

AT and its derivatives have evolved from traditional filler materials into key functional biomaterials and cellular sources in regenerative medicine. Their role in tissue repair and regeneration can be summarized systematically at three levels: as a composite graft of autologous cells and signaling factors; as a reservoir of multipotent stem cells and paracrine active factors; and as a core cellular component for advanced biofabrication technologies. The application of AT in tissue repair and regenerative medicine is progressing from empirical transplantation toward a new stage characterized by standardization, proceduralization, and precision. The core future challenges lie in establishing standardized operating procedures and quality control systems covering acquisition, processing, and application; and further optimizing ADSC‐based tissue engineering strategies, particularly addressing the vascularization issue in large‐scale tissue construction. By deepening the understanding of the immunomodulatory and regenerative mechanisms of AT and integrating cutting‐edge technologies from materials science, biofabrication, and cell biology, there is significant potential to fully exploit its clinical value as an autologous, multifunctional regenerative resource, achieving an important transition from simple filling to the regeneration of complex functional tissues.

### 5.2 Immune Dysregulation and Disease

AT is a highly heterogeneous immune microenvironment, with cellular composition and function varying significantly depending on anatomical location, which in turn determines its unique role in local and systemic diseases. When excessive nutrients are consumed, fat tends to accumulate in visceral and subcutaneous deposits, causing these reserves to enlarge due to hypertrophy and hyperplasia, and become unhealthy [192]. Unhealthy abdominal obesity is more detrimental to metabolic health, as it recruits macrophages and other immune cells, promotes systemic inflammation, and sustains ectopic fat accumulation [193]. The recruitment and proliferation of M1 macrophages are higher in visceral fat than in subcutaneous fat [194, 195, 196], while protective CD4⁺ helper cells and regulatory T cells (Tregs) decrease [197, 198], along with an enrichment of CD8⁺ T cells in visceral fat [199]. Abdominal obesity promotes insulin resistance and the development of type 2 diabetes, and is often accompanied by hypertension and dyslipidaemia; all of these factors are components of metabolic syndrome [200]. AT, particularly visceral fat, as a “factory” of inflammatory factors, releases inflammatory mediators (such as leptin, resistin, IL‐1β) that directly impair vascular endothelial function, promote plaque formation and instability, exacerbate oxidative stress, thereby accelerating the progression of atherosclerosis [201]. The epicardial AT surrounding the heart directly modulates coronary inflammation and atherosclerosis through paracrine actions, and its volume has become an independent predictor of cardiovascular risk [202]. Similarly, in the prostate cancer microenvironment, periprostatic AT (PPAT) actively drives tumor invasion and metastasis by secreting factors such as IL‐6 and CXCL12 and fostering a chronic inflammatory structure [203]. This spatial specificity suggests that interventions targeting specific adipose depots may represent a direction for future precision therapy. This spatial specificity suggests that interventions targeting specific fat stores may be the direction of future precision therapy. Fat accumulation is also regulated by epigenetic mechanisms. Obese fathers exhibit DNA methylation alterations in imprinted genes in human sperm, which may be inherited by their offspring [204]. Maternal dietary habits during pregnancy can modify DNA methylation in infants, influencing future fat deposition in children [205].

In summary, the imbalance of immune homeostasis in AT is a core pathophysiological link connecting obesity with a range of major chronic diseases. Its clinical significance is mainly reflected in four aspects: first, as a key driver of metabolic syndrome and cardiovascular disease; second, as a core component of tumor microenvironments in specific organs; third, revealing the potential for intergenerational transmission of disease risk; fourth, providing new directions for precise prevention and treatment strategies. Identifying circulating biomarkers for early diagnosis or risk prediction, for example, specific lipid metabolite profiles or ratios of adipose factors can predict cardiovascular events or diabetes risk. Understanding the core signaling pathways driving adipose dysfunction, such as insulin signaling and inflammatory pathways, provides a theoretical basis for developing novel metabolic disease drugs beyond GLP‐1 receptor agonists.

## 6 Conclusions

We reviewed the distribution of AT in different populations, its anatomical and physiological characteristics, as well as the roles of various components of AT in promoting repair and anti‐infection. Fat cells can directly participate in wound healing, assisting macrophages in resisting pathogen infections; the ECM secretes adhesion molecules and cytokines to regulate the proliferation and differentiation of precursor cells, promoting injury repair, epithelialization, and angiogenesis; stromal vascular components can activate macrophages, inhibit fibrosis, and promote wound healing and nerve repair; adipokines regulate multiple immune cells, promoting inflammation and local direct killing of invading microorganisms. This comprehensive and up‐to‐date overview of AT's roles in wound healing and anti‐infection opens new avenues for innovative therapies. It lays the foundation for the clinical application of AT and provides new strategies for the development of novel antimicrobial therapies.

### 6.1 Future Research Directions

For the immune function of AT, it is possible to delve into how various factors secreted by AT (such as leptin, adiponectin, and AMPs) play roles in specific immune responses, clarifying their functions under different inflammatory states or disease backgrounds. Researching the specific roles of different cellular components in AT (such as macrophages and ASCs) in immune responses can help understand their contributions to the onset and progression of diseases. Based on the biological functions of adipocytes and their factors, targeted drugs for specific factors can be developed to treat obesity‐related immune and metabolic disorders (such as type 2 diabetes and cardiovascular diseases). Utilizing stem cells and factors derived from adipose for tissue repair can promote the development of regenerative medicine. The functions of AT in the immune system hold broad prospects for research and application. Through in‐depth studies, targeted therapies, and comprehensive interventions, we can better understand its roles in health and disease, thereby providing new ideas and methods for improving human health (Table 1).

**Table 1:** Summary of literature on AT promoting repair and anti‐infection.

Composition of AT	Function	Future research directions and applications
Adipocytes	1. Adipocytes move to the wound site, cooperate with macrophages to remove debris cells; block the gap of the wound, prevent pathogens from entering [3]; 2. Adipocytes secret AMPs to directly kill pathogens [3]; 3. Adipocytes activate myofibroblasts and reprogram anti‐fibrotic [10, 54].	Whether and how adipocytes migrate to wounds in mammals.
ECM	1. ECM secretes a variety of adhesion factors and growth factors [36, 58]; 2. ECM regulates the proliferation, differentiation, and maturation of adipose precursor cells [36]; 3. ECM increases vascular distribution and collagen deposition [59, 60].	It can be combined with stem cell tissue engineering technology to repair the damaged parts.
Immunocyte		
Macrophage	1. M1 macrophages produce pro‐inflammatory factors (TNF‐α, IL‐6) [73, 74]; 2. M2 helps tissue repair and is anti‐inflammatory [71].	Macrophages, T cells, and B cells in AT participate in the role and mechanism of wound repair.
T cells	1. Secretion of IL‐17 regulates DNL [78, 79, 80]; 2. IL‐17A signaling participates in controlling local parasite burden [83]; 3. Secretion of cytokines: IFN‐γ promotes M1 polarization; IL‐4, IL‐13, and IL‐10 promote M2 polarization [70, 87]; 4. iNKT cells inhibit macrophage infiltration in AT [88, 89].	
B cells	1. CD5⁺ B‐1a cells secrete IL‐10 to improve cardiac function and injury repair [93]; 2. IgG produced by B cells regulates Breg‐secreted IL‐10, which suppresses AT inflammation [98].	
SVF		
ADSC (the cell with the strongest regenerative ability in SVF)	1. Proliferation and differentiation of damaged cells to promote repair [105, 112]; 2. Activate cell regeneration, promote collagen deposition, reduce inflammatory response to accelerate the healing process [128]; 3. Promote the formation of new blood vessels (differentiation into endothelial cells, release of angiogenic factors) [128, 130, 131, 132]； 4. It is the main source of participation in maintaining ECM proteins [126].	Based on its advantages, it can be used as a seed cell in tissue engineering to develop new clinical treatments.
SVF (ADSC + all other cells)	1. Activate macrophages and reduce inflammatory cell infiltration [11]; 2. Secretion of IL‐10 inhibits fibrosis [104, 106]; 3. Activation of TLRs/MyD88/NF‐κB pathway promotes wound healing and nerve repair in diabetes [67].	
Adipokines		
Leptin	1. Leptin induces TNF‐α and IL‐6 production, enhances the phagocytosis of macrophages [139]; 2. In the face of *Staphylococcus aureus* infection, macrophages will regulate leptin production and regulate angiogenesis [141]; 3. In vitro, it can activate PI3K and MAPK signaling pathways to block apoptosis [143].	An in‐depth study of the role of adipokines in immune response lays the foundation for the development of new infection treatment strategies.
Adiponectin	1. Activation of NF‐κB and ErK pathways to upregulate pro‐inflammatory factors (TNF‐α, IL‐6, and IL‐8) to promote inflammatory response [148, 149, 150]; 2. Participate in parasite removal [151].	
AMPs	1. Bacteria and fungi activate Toll and IMD pathways, leading to the secretion of AMP [158]; 2. The fat body of the fruit fly can secrete AMPs to resist pathogen invasion [3]; 3. Mouse adipocytes can produce AMP after bacterial infection [9]; 4. Defensins and cathelicidins exhibit chemotactic properties and can recruit immune cells to sites of infection [152, 153].	Few studies have been conducted on the secretion of AMP by AT in vertebrates. An in‐depth study of the mechanism that promotes AMP secretion is of great significance for the anti‐infection effect of AT.

## Author Contributions

Xi Duan conceived this review and participated in its design. Run Li conducted manuscript writing. Xi Duan revised the manuscript, Lei Fu made the diagram, Jiali Yang and Zhean Zhan are responsible for the revision of the review. All authors have read and approved the final manuscript.

## Funding

The authors received no specific funding for this work.
